# Eliciting pyroptosis to fuel cancer immunotherapy: mechanisms and strategies

**DOI:** 10.20892/j.issn.2095-3941.2022.0049

**Published:** 2022-07-21

**Authors:** Wuyin Wang, Lu Zhang, Zhijun Sun

**Affiliations:** 1 The State Key Laboratory Breeding Base of Basic Science of Stomatology (Hubei-MOST) & Key Laboratory of Oral Biomedicine Ministry of Education, School & Hospital of Stomatology, Wuhan University, Wuhan 430079, China; 2 Department of Oral Maxillofacial-Head Neck Oncology, School & Hospital of Stomatology, Wuhan University, Wuhan 430079, China

**Keywords:** Pyroptosis, immune checkpoint blockade, immunogenic cell death, tumor, immunotherapy

## Abstract

Immune checkpoint blockade (ICB) therapy has recently shown promise in treating several malignancies. However, only a limited number of patients respond to this treatment, partially because of the “immune cold” condition of the tumor immune microenvironment. Pyroptosis is a type of gasdermin-mediated programmed cell death that often leads to inflammation and immune responses. Many studies on the mechanism and function of pyroptosis have led to increasing recognition of the role of pyroptosis in malignant progression and immune therapy. Pyroptosis has the potential to alter the tumor immune microenvironment by releasing tumor-associated antigens, damage-associated molecular patterns, and proinflammatory cytokines, thus leading to intratumoral inflammatory responses, stimulation of tumor-specific cytotoxic T cell infiltration, conversion of “cold” to “hot” tumors, and ultimately improving the efficacy of ICB therapy. Some cancer treatments have been shown to restore anticancer immunosurveillance through the induction of pyroptosis. Therapy promoting pyroptosis and ICB therapy may have synergistic effects in cancer treatment. This review summarizes the mechanisms and roles of pyroptosis in the tumor microenvironment and combination treatment strategies. An improved understanding of the roles of pyroptosis in tumorigenesis, immune evasion, and treatment would aid in the development of therapeutic strategies for malignancies.

## Introduction

Malignant neoplasms are a major global health concern placing a massive burden on society worldwide^1,2^. The need for development of effective therapeutic and diagnostic strategies is exceptionally urgent. Conventional cancer treatment modalities, such as surgery, chemotherapy, radiotherapy, and targeted therapy, directly affect tumors by killing cancer cells, preventing cancer cells proliferation, inducing mutations and apoptosis. However, the immune system, a highly influential tumor-extrinsic factor, had not received widespread attention until the development of modern immune checkpoint blockade (ICB) immunotherapy. This novel treatment with immune checkpoint inhibitors (ICIs) achieves substantial efficacy by enhancing the function of antitumor T cells, and was the topic of the 2018 Nobel Prize. Although ICB treatment has provided encouraging results and advanced treatment concepts in oncology, few patients respond to checkpoint inhibitor drugs^3–5^. The lack of response to treatment in many patients has become an obstacle in cancer immunotherapy.

**Figure 1:**
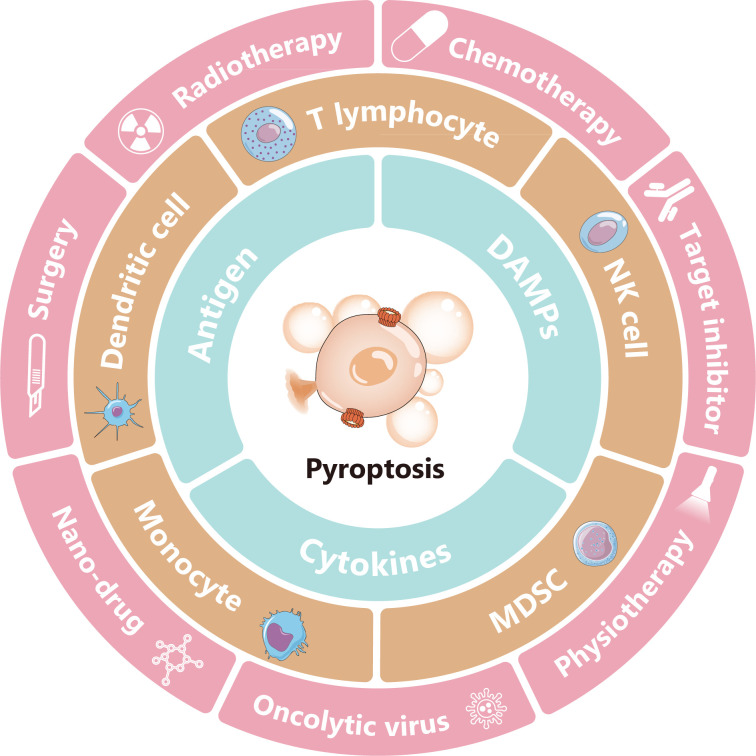
Pyroptosis and cancer therapy. Pyroptosis is lytic and highly pro-inflammatory cell death. Promotion of pyroptosis can directly kill tumor cells. Remarkably, pyroptotic cancer cells produce and release DAMPs, antigens, and pro-inflammatory chemokines, thereby regulating the proportions of tumor-infiltrating immune cells such as T cells, NK cells, DCs, monocytes, and MDSCs; changing the tumor immune microenvironment; and changing “cold” tumors to “hot” tumors. In most cases, this process promotes anti-tumor immunity. However, pyroptosis may result in adverse effects to normal tissue and even promote tumor progression. Surgery, radiotherapy, chemotherapy, target inhibitor, nano-drug treatment, oncolytic viruses, and physiotherapy, such as photodynamic therapy, cause varying degrees of cell pyroptosis. The utilization of pyroptosis, particularly in combination with ICB treatment, is a promising cancer treatment.

Inducing pyroptosis may increase the response rate to ICB by activating the host’s immune system. Pyroptosis is a type of gasdermin (GSDM)-mediated programmed cell death (PCD) characterized by cell swelling with large bubbles, rupture, release of cellular contents, and necrotic cell death, which often lead to inflammation and immune responses^6^. In contrast to immunologically silent apoptotic cells, pyroptotic cells rapidly release cellular contents, including cell antigens, proinflammatory cytokines, and damage-associated molecular patterns (DAMPs), thus influencing the inflamed tumor immune microenvironment, activating host antitumor immunity, and potentially leading to tumor regression^6–8^. Proinflammatory cytokine production in pyroptotic cells can also contribute to antitumor immunity. In addition, pyroptosis-induced signals activate antigen-presenting cells (APCs), causing them to enter a “hyperactive” state characterized by enhanced migration and stimulation of stronger cytotoxic T lymphocyte responses^9,10^. The role of pyroptosis in cancer therapy is shown in **Figure 1**.

Promoting inflammatory cell death, such as ferroptosis, necroptosis, and pyroptosis, has recently been proposed to stimulate the host immune system to exert antitumor effects^11–13^. However, rapid developments in basic research in recent years have provided a new understanding of the molecular mechanisms of pyroptosis. The emergence of novel antitumor strategies, such as nanomaterials, physiotherapy, and oncolytic viruses, has increased the number of methods available to fight cancer, thus necessitating a new understanding of pyroptosis and the design of appropriate clinical treatment strategies to enhance the effectiveness of immunotherapy and maximize patient benefit. Demonstrating both mechanisms and strategies would bridge basic and translational oncology research, and enable the development more effective therapeutic strategies. This review summarizes the molecular mechanisms of pyroptosis and combination treatment strategies with immunotherapy. Pyroptotic cancer cells actively induce the immune response in the tumor immune microenvironment by releasing tumor-associated antigens, DAMPs, and proinflammatory cytokines, which in turn stimulate tumor-specific cytotoxic T cell infiltration and enhance antitumor immunity. The exploitation of pyroptosis, particularly when combined with ICB, is a promising cancer treatment. Extensive exploration of the role of pyroptosis in cancer immunity, and of rational combination strategies with ICB and localized pyroptosis-inducing therapies, are necessary to improve patient prognosis.

## Mechanism of pyroptosis

**Figure 2:**
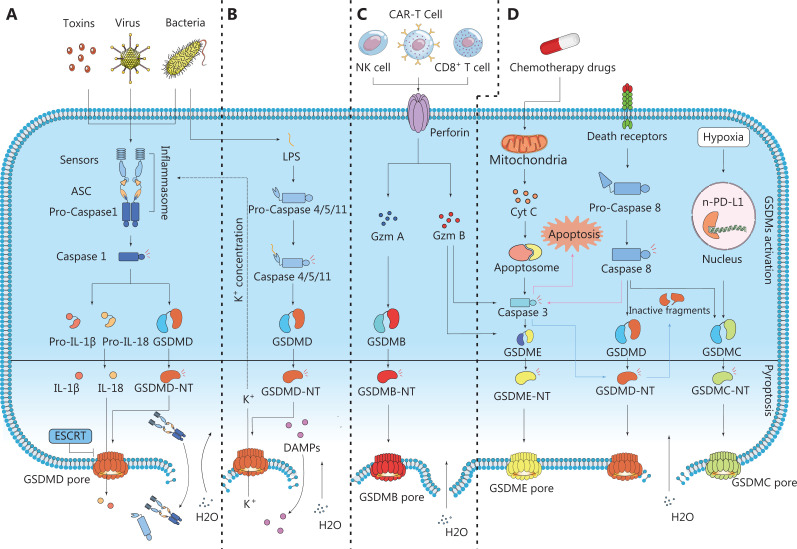
Schematic diagram of pyroptosis pathways. The process of cell pyroptosis can be divided into 2 broad steps: GSDM activation and pyroptotic cell death. The first step includes several routes for active GSDMs to form nonselective pores in the plasma membrane and lead to cell death. (A) Canonical pathway. In the canonical pathway, upstream stimulation signals lead to inflammasome formation and activation of caspase-1. Activated caspase-1 cleaves GSDMD and pro-IL-1β and pro-IL-18, thus leading to the pore-forming activity of the GSDMD-NT and the activation of IL-1β and IL-18. GSDMD-NT forms nonselective pores in the plasma membrane; initiates cell swelling with large bubbles, rupture, and the release of cell contents; and subsequently leads to cell death. (B) Non-canonical pathway. Caspase-4/5/11 directly recognizes LPS through the CARD domain, thus triggering oligomerization and activating proteolytic activity. Activated caspase-4/5/11 cleaves GSDMD and results in pyroptosis. The IL-1β/18 exudation relies on the activation of the canonical pathway. (C) Recent reports have suggested that Gzm A and Gzm B also induce pyroptosis through cleavage of GSDME or GSDMB. (D) Other pathways. Some molecules in apoptosis, such as caspase-3 and caspase-8, have also been shown to be involved in pyroptosis. GSDME is the substrate for caspase-3 when it induces pyroptosis, and caspase-8 cleaves GSDMD and GSDMB. Caspase-3 also decreases pyroptosis by cleaving GSDMD at ASP87, thereby eliminating pore formation. In hypoxia, nuclear PD-L1 (nPD-L1) enhances the transcription of the GSDMC gene and directly regulates the caspase-8/GSDMC pyroptosis pathway. Remarkably, the ESCRT machinery may be involved in repairing membrane damage caused by GSDM activation.

Pyroptosis, a type of cell death, was named in 2001. *Pyro* comes from the Greek root meaning fever or relating to fire. *Ptosis* means falling, and it describes a proinflammation PCD^14^. At that time, pyroptosis was defined as caspase-1-dependent cell death. In 2015, gasdermin D (GSDMD) was reported to be an executioner in pyroptosis^15^. With the discovery of GSDMs^16^, inflammasomes^17^, and the noncanonical pyroptosis pathway^18^, pyroptosis has been redefined as GSDM-mediated programmed necrotic cell death^6,19^. The association between pyroptosis and cancer therapy has received increasing attention. The mechanism of pyroptosis is briefly summarized in the sections below (**Figure 2**).

### Canonical pathway

Canonical pyroptotic cell death is mediated by caspase-1 and executed by GSDMD. Upstream stimulation signals lead to inflammasome formation and the activation of caspase-1. The inflammasome is a macromolecular complex consisting of 3 essential components: NOD-like protein (NLR), apoptosis-associated speck-like protein (ASC), and pro-caspase-1. NLRs are a type of pattern-recognition receptor that recognizes pathogen-associated molecular patterns on pathogens, and DAMPs on host or tumor tissue^20^. When subjected to exogenous or endogenous stimulation, NLR proteins bind ASC, an adaptor molecule containing a caspase activation and recruitment domain. Activated inflammasome sensors nucleate ASCs, which in turn recruit pro-caspase-1, thereby forming the complete inflammasome, which induces self-cleavage of caspase-1. Activated caspase-1 cleaves GSDMD, thus relieving the autoinhibitory activity of the C-terminal domain and leading to pore-forming activity of the N-terminus of GSDMD (GSDMD-NT)^6,21^. GSDMD-NT forms nonselective pores in the plasma membrane; initiates cell swelling with large bubbles, rupture, and cell content release; and subsequently leads to cell death^22,23^. Pro-IL-1 and pro-IL-18 are processed into mature forms by activated caspase-1 and are released from GSDMD pores in this process^6^.

Remarkably, new evidence has shown that GSDMD pore formation is reversible. Calcium influx through membrane pores triggers membrane repair by the endosomal sorting complexes required for transport (ESCRT) machinery. ESCRT-III removes the GSDMD pores, thereby reversing pyroptosis^24,25^. Activation of GSDMD in some cases causes cells to advance to a “hyperactive” stage and release IL-1β^26^. The effects of hyperactive cells are described in detail in later sections.

### Noncanonical pathway

After the discovery of canonical pyroptosis mediated by caspase-1, caspase-11 was found to be involved in pyroptosis. Caspase-4 and caspase-5 are homologs of caspase-11 in humans. In contrast to caspase-1, caspase-4/5/11 directly recognize LPS through the caspase recruitment domain (CARD), and subsequently trigger oligomerization and the activation of proteolytic activity^18,27^. Activated caspase-4/5/11 cleaves GSDMD and results in pyroptosis^21,28^. Additionally, another study has indicated that caspase-11 cleaves pannexin-1 channels and releases adenosine triphosphate (ATP). ATP activates P2X ligand-gated ion channel (P2X7) receptors, thereby mediating pyroptosis^29^. Interestingly, caspase-4/5/11 cleaves GSDMD, similarly to caspase-1, but cannot process pro-IL-18 or pro-IL-1β. After initiation of pyroptosis by caspase-4/5/11, the nod-like receptor family pyrin domain containing 3 (NLRP3) inflammasome is activated by potassium efflux and subsequently induces IL-1β/18 release^30–32^. This process may be caused by GSDMD pore formation^6,33^ or pannexin-1 activation^29^.

### Other pathways

Caspases -3 and -8 were first recognized as the initiators in extrinsic apoptosis, an immunologically silent form of cell death. Subsequent studies have proven they are also involved in cellular pyroptosis^34^. GSDME is the substrate for caspase-3 when it induces pyroptosis. After chemotherapy, GSDME is cleaved by caspase-3 after ASP270, thus generating an GSDME-NT that perforates the cell membrane and causes pyroptosis. The mechanism is also associated with adverse effects of chemotherapy^35^. Notably, caspase-3 and caspase-7 also decrease pyroptosis by cleaving GSDMD at ASP87, thereby eliminating pore formation^36^.

Additionally, therapeutics designed for cancer chemotherapy have been found to promote caspase-8/9-dependent pyroptosis, as well as NLRP3 inflammasome activation, which is associated with the channel-forming membrane protein pannexin-1^37^. Furthermore, caspase-8 has been found to regulate apoptosis, necroptosis, and pyroptosis^38^. In the hypoxic environment, p-Stat3 interacts with nuclear PD-L1 and enhances transcription of the *GSDMC* gene. Caspase-8 induces pyroptosis by cleaving GSDMC, thereby resulting in a switch from TNFα-induced apoptosis to pyroptosis^37^.

Other molecules beyond caspases may be responsible for cleaving GSDM in the regulation of pyroptotic cell death. A previous study on chimeric antigen receptor (CAR) T cell therapy has demonstrated that granzyme B (Gzm B) from CAR T cells activates the caspase-3/GSDME pathway and induces pyroptosis by hydrolyzing caspase-3. The release of cellular contents in pyroptosis activates caspase-1 for GSDMD cleavage in macrophages. These pyroptotic macrophages subsequently release cytokines, thereby resulting in hydrolysis^39^. Gzm B also targets cell pyroptosis by directly cleaving GSDME at the same site as caspase-3, thus enhancing antitumor immunity^40^. To kill cells through pyroptosis, granzyme A (Gzm A) from NK cells and cytotoxic T lymphocytes cleave GSDM B (GSDMB), a pyroptotic executor protein in the GSDM superfamily.

### Crosstalk among apoptosis, necroptosis, and pyroptosis

**Figure 3:**
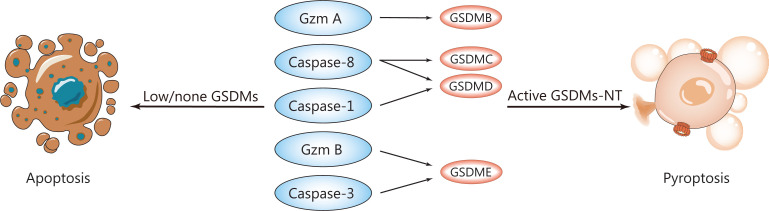
Essential molecules in pyroptosis. GSDMs, cleaved by several proteinases for activation, are the executioners of pyroptotic cell death. The important GSDMs inducing pyroptosis and their upstream proteinases are listed. GSDMB is cleaved by granzyme A into the active forms GSDMC by caspase-8; GSDMD by caspases -1 and -8; and GSDME by caspase-3 and granzyme B. Caspase-8 indirectly activates GSDME by cleaving caspase-3. Additionally, these proteinases have the potential to induce apoptosis when GSDMs are not expressed or weakly expressed.

The substantial crosstalk observed between pyroptosis and apoptosis pathways suggests that the activation or repression of critical proteins promotes the switch from apoptosis to pyroptosis, and subsequently enhances antitumor immunity^41^. Multiple GSDMs are differentially activated by distinct caspases or granzymes in various pyroptotic pathways. The caspase or granzyme functions are of interest as molecular switches, because of the relationship between apoptosis and pyroptosis in cells (**Figure 3**).

The concept of “PANoptosis” has recently been proposed to highlight the crosstalk and coordination among pyroptosis, apoptosis, and necroptosis^42^. Numerous critical molecules in these pathways interact in a molecular scaffold called the “PANoptosome,” and subsequently trigger and regulate specific cell death^43,44^. Moreover, Z-DNA-binding protein 1 (ZBP1)-mediated PANoptosic cell death has been found to inhibit tumorigenesis in mice^45^. Further studies may elucidate the mechanistic features and enhance immunotherapeutic efficacy. Rational design of therapeutic strategies is necessary to investigate the crosstalk among apoptosis, pyroptosis, and other forms of cell death in the cancer immune response.

## Pyroptosis of cancer cells regulates the tumor immune microenvironment

### Pyroptotic tumor cells release DAMPs and proinflammatory cytokines, thus affecting the TME

Pyroptosis induces inflammation through the release of DAMPs and inflammatory cytokines. Typical cellular contents include ATP, HMGB1, and IL-1 family cytokines. These DAMPs and cytokines modulate the innate immune response, enhance adaptive immunity, and increase antigen presentation and TLR activation^46^.

ATP and DAMPs released during pyroptosis are sufficiently small to cross the GSDMD pores, thus these small molecules start to be released before cytolysis^47^. Extracellular ATP released by pyroptotic cells activates P2X7 receptors, thereby forming new membrane pores that release DAMPs and inflammatory cytokines. In addition, the canonical NLRP3 inflammasome-associated pyroptosis pathway is activated by pannexin-1-mediated potassium efflux^29,48,49^. These results suggest that an ATP-associated positive feedback loop may exist during pyroptosis. ATP operates as a “find me” signal that recruits monocytes, macrophages, and dendritic cell (DC) precursors, and subsequently leads to the clearance of dying cells^19,50,51^. ATP released by dying pyroptotic cancer cells also activates DCs by engaging DC P2X7 receptors and then priming T lymphocytes, which provide effective antitumor immunity^52^.

High-mobility group box-1 (HMGB1) is a 25 kDa nuclear protein that is too large to pass through GSDMD pores and thus is released after cellular rupture^53^. HMGB1 acts as a DAMP by binding pattern recognition receptors after being released into the extracellular milieu^54^. The complex functions of HMGB1 are likely to be associated with its oxidation state, in which different receptors can combine and activate different downstream pathways in the tumor microenvironment (TME). Newly released HMGB1 in a fully reduced form (fr-HMGB1) can be oxidized to disulfide-HMGB1 (ds-HMGB1) in the oxidative environment. HMGB1 can also be sulfonated by reactive oxygen species (ROS), thus forming ox-HMGB1^55^. Fr-HMGB1 and CXCL12 form a heterocomplex that induces the recruitment of inflammatory cells^56,57^. Ds-HMGB1 displays cytokine-stimulating activity that promotes inflammation through TLR4/myeloid differentiation factor 2 (MD-2)^58^. The chemoattractive and proinflammatory activity are entirely suppressed in ox-HMGB1. During apoptosis, caspase targets the mitochondria, causing them to produce ROS, which oxidizes HMGB1 and promotes immune tolerance^59^. In contrast, pyroptotic cells that release fr-HMGB1 induce a powerful antitumor immune response^60^.

IL-1β and IL-18 lack classical peptides for protein secretion and are secreted through GSDMD pore formation during pyroptosis^61^. The secretion dose does not rely on cell lysis. After a specific stimulus, the macrophages and DCs can achieve a state of hyperactivation that can release IL-1 while retaining viability^9,62^. The ESCRT-III machinery for plasma membrane repair may play an essential role in the hyperactive state^24^. IL-1β and IL-18 have dual roles in the TME and distal tissues, and exert varying effects at different stages of tumor development. As levels of IL-1β increase, the number of myeloid-derived suppressor cells (MDSCs) and Treg cells increase accordingly^63,64^. However, IL-1β also enhances the anti-cancer ability of tumor-specific T cells in B16 melanoma tumors in mice^65^. One recent study has indicated that IL-18 enhances the therapeutic effects of anti-PD-1 and CTLA-4 ICB therapy^66^.

### Pyroptosis induces a strong immunogenic response in cancer

#### APCs amplify inflammatory signals during pyroptosis

**Figure 4:**
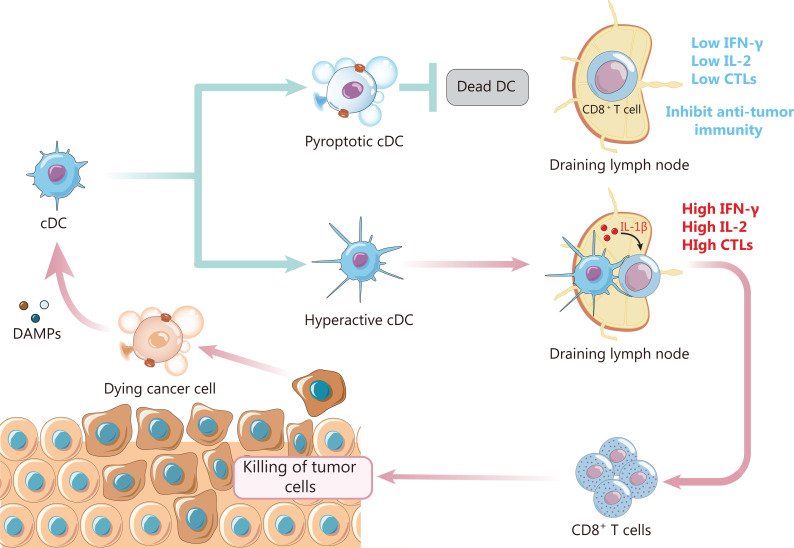
APCs amplify inflammatory signals during pyroptosis. Pyroptotic cancer cells may provide signals including tumor antigens, DAMPs, and pro-inflammatory cytokines, which activate naïve T cells. This process relies on APCs. Interestingly, inflammasome activation in DCs may lead to DC pyroptosis or a "hyperactive" state. Pyroptotic DCs lead to the disruption of T cell activation signals, thus hindering an anti-tumor immune response, but DCs in the "hyperactive" state have a stronger ability to migrate to draining lymph nodes, and can continue to release pro-inflammatory cytokines and activate T cells more effectively. The process amplifies inflammatory signals during cancer cell pyroptosis, thus triggering a strong anti-tumor immune response.

Pyroptotic cells enhance the adaptive immune response by releasing tumor antigens, DAMPs, and proinflammatory cytokines. APCs are recruited by pyroptotic cells, which release ATP. Phagocytic uptake of pyroptotic cells leads to maturation of DCs and cross-presentation to T cells^46^. Pyroptotic cells expose antigen-like filamentous actin (F-actin), which may be accessible to DCs to cross-presentation CD8 T cells. IL-1β and IL-18 derived from pyroptotic cells may promote DC maturation^8^. Overall, pyroptosis releases inflammatory mediators, stimulates DC maturation, and activates CD8 T cells, thus leading to a strong immune response. Moreover, recent research has shown that inflammasome activation in phagocytes induces the secretion of IL-1β by cells, which remain viable^67^. This state is called “hyperactive” (**Figure 4**). DCs, macrophages, and neutrophils have been demonstrated to achieve this state. Compared with naïve or traditionally active DCs, hyperactive DCs display highly extended membrane protrusions, and show strong migration and directionality when the motility of single cells is tracked. They can also immigrate to draining lymph nodes, thereby triggering a more powerful and durable anti-tumor immune response^10^.

#### T cell response to pyroptosis

Numerous experiments have demonstrated that pyroptosis increases T cell infiltration in tumors, enhances T cell function, and promotes antitumor immunity. Early research has indicated that the IL-1β source of NLRP3 activation is necessary to cross-prime the antitumor CD8 T cell response^52^. In addition, IL-1β increases the population size and enhances the antitumor ability of adoptively transferred T cells in mice^65^. IL-1R is expressed on the surfaces of naïve and memory T cells. IL-1β promotes naïve T-cell polarization into T helper cells and transformation into CD8 T cells^67^. GSDME-mediated pyroptosis has been found to increase T cell infiltration and improve therapy responses in a melanoma model^68^. Less than 15% of tumor cell pyroptosis has been found to clear the entire 4T1 graft in a mammary cancer model. Athymic^Nu/Nu^ mice lacking mature T cells do not display tumor regression, given that T cells play a decisive role in this regression^69^.

## Pyroptosis and cancer therapy

### Surgery

Conventional treatments, such as surgery, radiotherapy, and chemotherapy, are widely used for the clinical treatment of various tumors. Traditional oncological treatments have long been believed to directly kill cancer cells. Recently, the roles of conventional therapies in the tumor immune microenvironment have attracted considerable attention^70^. The relationship between surgery and pyroptosis has only recently been discovered.

Surgical resection has a long history and is the primary treatment option for many solid cancers. The aim of resection is to remove all macroscopic tumor tissue and lymph node involvement. Recent research has suggested that surgical treatment favors immune suppression^71^. Damaged tissue releases DAMPs into the TME and the circulation, the latter of which is associated with resistance suppression^72^. These DAMPs may lead to the formation of an inflammatory environment through the pyroptotic pathway and recruit immunosuppressive cells such as MDSCs, M2 macrophages, or Tregs^71^. Inhibition of pyroptotic pathways activated by surgery may promote the tumor immune response.

### Radiotherapy

Radiotherapy and chemotherapy are traditionally used to kill rapidly dividing cells during cancer treatment. Many patients with cancer receive radiotherapy or chemotherapy. Pyroptosis has been shown to play an essential role in cancer treatment^70,73^. High expression of GSDME in colorectal cancer cells enhances the effects of radiotherapy. Radiotherapy kills cancer cells by directly damaging DNA. Recognition of the resultant DNA fragments by the AIM2 receptor subsequently activates the AIM2-ASC-caspase 1 inflammasome, thus leading to the secretion of IL-18 and IL-1β, and cell pyroptosis^74^. The DNA damage caused by radiotherapy also elicits immunogenic effects that enhance antitumor immunity^46^. In the tumor immune environment, ionizing radiation also increases the concentrations of proinflammatory cytokines by inducing macrophage pyroptosis^75^. These processes can change immunologically “cold” tumors to “hot” tumors, and benefit tumor therapy.

### Chemotherapy

Chemotherapy has also been shown to induce pyroptosis. Many chemotherapeutic drugs cause pyroptosis in cancer cells expressing high levels of GSDME. This process relies on the activation of caspase-3^76^. However, pyroptosis caused by chemotherapeutic drugs is considered a double-edged sword for patients with tumors. On the one hand, pyroptosis effectively activates host anti-tumor immunity, but on the other, normal tissue pyroptosis triggered by chemotherapeutic drugs leads to chemotherapy side effects. GSDME is highly expressed in most normal tissues and weakly expressed in some malignant tumors. Fewer adverse effects of chemotherapeutic drugs have been observed in GSDME^-/-^ mice than in wild-type mice^76^. Epigenetic drugs have a notable role in pyroptosis. As previously described, the expression of several GSDM molecules is decreased by epigenetic alterations in cancer cells^77^. Decitabine (DAC), one of the most commonly used epigenetic drugs, was developed to enhance the therapeutic efficacy of chemotherapy. Pretreatment of tumor-bearing mice with DAC increases chemosensitivity by promoting GSDME-mediated pyroptosis^78^. Another DNA methyltransferase inhibitor, γ-oryzanol, upregulates the expression of GSDMD in cancer cells, thus enhancing cancer immunotherapy in animal models^79^. The main remaining question in exploiting these proinflammatory effects is how to increase the efficacy of immunotherapy, and decrease or avoid adverse effects.

### Nanodrug and physiotherapy

Many small-molecule chemotherapy drugs, such as cisplatin, paclitaxel, and doxorubicin, induce cancer cell pyroptosis. However, they are limited in cancer therapy because of their nonspecific biodistribution and systemic adverse effects. Nanotechnology is expected to address these issues. Many pyroptosis-based nanodrugs have been developed^80^. Various nanomaterials can address the drawbacks of pyroptotic reagents by facilitating tumor accumulation and minimizing adverse drug reactions^81^. Some physical therapy modalities, such as photodynamic therapy, have been combined with nanodrugs as biomimetic nanoparticles to induce pyroptosis^82^. The photoactivated pyroptosis mediated by biomimetic nanoparticles induces systemic antitumor immunity and suppression of tumors^83,84^. These nanodrugs, which have the advantages of specific biodistribution, prolonged blood circulation times, and controlled drug release, hold promise for cancer therapy.

### Targeted inhibitors

Target therapy has been demonstrated to increase the therapeutic effects of immunotherapy by reversing the immunosuppressive TME^85^. Several targeted drugs designed to block critical pathways in tumor survival and progression also positively regulate the effects of pyroptosis. In a melanoma study, a combination of BRAF and MEK inhibitors (BRAFi + MEKi) has been found to cause pyroptosis through the caspase-3/GSMDE pathway^68^. Similar results have been observed with BI2536, a PLK1 kinase inhibitor, and DDP combination treatment in GSDME-overexpressing esophageal squamous cell carcinoma^86^. In addition, several small-molecule inhibitors targeting KRAS, EGFR, and ALK in lung cancer also stimulate cancer cell pyroptosis through the caspase-3/GSDME pathway^87^.

Another commonly used target is CD39/CD73. CD39 degrades ATP to AMP, and is mainly expressed on endothelial cells and Treg cells. CD73 is expressed on the surfaces of T cells and B cells, and it degrades AMP to adenosine. Pyroptotic cancer cells release ATP to promote antitumor immunity, but under the synergistic effects of CD39 and CD73, ATP is eventually converted into immunosuppressive extracellular adenosine^88^, thereby negatively affecting antitumor immunity. Inhibitors of CD39/CD73 can be applied alone or in combination to block this process. CD39/CD73-targeting agents have recently entered clinical trials. More recently, we have engineered a bioresponsive nanoparticle that effectively enhances the efficacy of anti-PD-L1 therapy through synergistic effects of pyroptosis and CD73 inhibition^79^.

### Oncolytic viruses and bacteria

Oncolytic viruses kill cancer cells through oncolysis and elicit antitumor immunity^89^, the latter of which functions by inducing pyroptosis or other immunogenic cell death. Lysed cells release DAMPs, ILs, and tumor-associated antigens, thus activating DCs and eliciting antitumor immunity. Some clinical trials on oncolytic viruses combined with ICB in cancer have been reported. Antibody-targeted ICIs enhance the efficacy of oncolytic virotherapy^90^.

Additionally, intratumor bacteria have been detected in various cancers and found to correlate with the response to immunotherapy. Tumor-targeting bacteria have also been engineered to fight cancer^91,92^. Overall, studies have illustrated the potential therapeutic strategies for inducing pyroptosis in cancer, thus indicating possibilities for combination therapies with ICIs.

## Potential for synergy between immune checkpoint blockade and localized pyroptosis-inducing therapies

**Figure 5:**
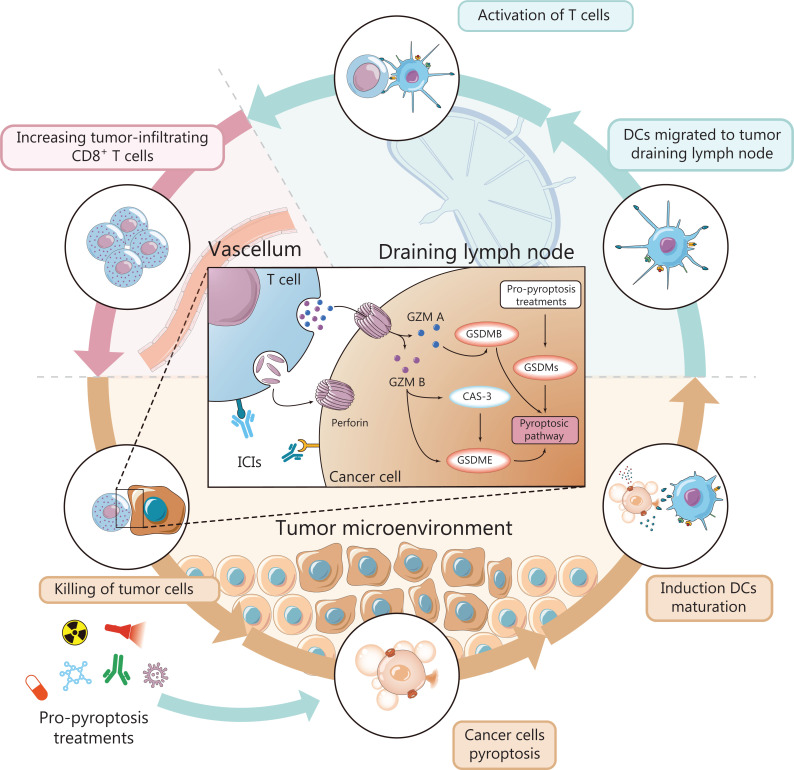
Pyroptosis-inducing therapies enhance the efficacy of ICB therapy. Pyroptotic cancer cells may promote T cell maturation and migration, and anti-tumor effects by releasing DAMPs, tumor antigens, and cytokines. Increasing numbers of CD8^+^ T cells lay the groundwork for ICIs. Furthermore, the granzyme released by CD8^+^ T cells increases tumor pyroptosis, thus creating a positive feedback loop for tumor immunotherapy. Overall, combining localized pyroptosis-inducing therapy and ICB may potentially augment the therapeutic efficacy of immunotherapy.

ICB-based therapeutic strategies are promising for cancer therapy. Many negative and positive checkpoints have been discovered and have advanced to preclinical/clinical testing^93^. Although these developments offer hope to some patients with malignancies, many patients do not respond to this treatment. The success of ICB strategies relies on activating antitumor T cells and is dependent on the appropriate TME^94,95^. Pyroptosis increases the immunogenicity of cancer, forms a proinflammatory tumor immune microenvironment, warms “cold” tumors, and recruits antitumor T cells. Pyroptosis also affects other immune cells, such as macrophages and DCs, thus influencing tumor immunity. Hence, ICB therapy and pyroptosis inducers might potentially be used in combination to stimulate synergistic effects in cancer therapy (**Figure 5**).

As described above, many chemotherapy drugs have the potential to cause pyroptosis^40,69^. In addition to the chemotherapy drugs approved in clinical therapy, some preclinical compounds induce cancer cell pyroptosis (**Table 1**). Animal experiments have indicated that less than 15% of 4T1 mammary cancer cell pyroptosis is sufficient to eliminate whole tumors through activating anti-cancer immunity^69^. Nanodrugs have also shown good application prospects. In a recent study, Xiao et al.^112^ designed a tumor-specific pyroptotic inducer that can be released in the tumor immune microenvironment. Under chemo-photodynamic therapy, the nanoprodrug induces tumor cell pyroptosis and boosts PD-1 treatment efficiency. It can also prevent tumor recurrence by generating immunological memory.

**Table 1:** Clinical drugs and pre-clinical compounds that activate pyroptosis in cancer cells

Name of drug	Target protein	Gasdermin	Cell types	Daily use	Reference
Docosahexaenoic acid	NLRP3	GSDMD	Breast cancer	Nutrient	^96,97^
Cisplatin	Caspase-3	GSDME	Lung cancer, esophageal squamous cell carcinoma	Chemotherapy	^98,88^
Paclitaxel	Caspase-3	GSDME	Lung cancer	Chemotherapy	^98^
As_2_O_3_	NLRP1, 3	GSDME	Hepatocellular carcinoma	Chemotherapy	^99^
Iron	Tom20	GSDME	Melanoma	Anti-anemia	^100^
Doxorubicin	eEF-2K	GSDME	Melanoma	Chemotherapy	^101^
Pyridoxine	Caspase-3	GSDME	leukemia	Nutrient	^102^
Lobaplatin	JNK	GSDME	Colon cancer	Chemotherapy	^103^
Metformin	PELP1	GSDMD	Esophageal carcinoma	Anti-diabetes	^104^
Anthocyanin	NLRP3	GSDMD	Oral squamous cell carcinoma	Nutrient	^105^
DPP 8/9 inhibitors	NLRP1, CARD8	GSDMD	Leukemia	-	^106,107^
FL118	NLRP3	GSDMD	Colorectal cancer	-	^108^
α-NETA	Pro-caspase-4	GSDMD	Epithelial ovarian cancer	-	^109^
L61H10	Caspase-3	GSDME	Lung cancer	-	^110^
Miltirone	MEK, ERK	GSDME	Hepatocellular carcinoma	-	^111^

Combining therapy with targeted therapy that induces pyroptosis and ICB is also an attractive option. In melanoma, the combination of BRAFi and MEKi strengthens antitumor immunity by inducing pyroptosis. In tumor-bearing BRAFi- and MEKi-resistant mice, etoposide induces renewed pyroptosis, slows tumor growth, and increases survival^68^. More interestingly, PD-L1, a major molecule used in ICB therapy, is involved in pyroptotic regulation. PD-L1 is generally expressed on the cell membrane, although it can translocate into the nucleus or be secreted to the outside of the cell^113^. Nuclear PD-L1 enhances the transcription of the GSDMC gene and directly regulates the caspase-8/GSDMC pyroptosis pathway^114^ (**Figure 1**).

The success of animal studies suggests that similar therapeutic strategies may aid in clinical oncology. Although many of the drugs listed in **Table 1** are not highly effective against cancer when used alone, given their pyroptosis-inducing effects, combining them with ICB therapy is likely to increase the success of ICB therapy and result in good patient prognosis. A variety of combination treatment strategies are now being tested in clinical trials, and the results have shown that combination therapy is more effective than ICB treatment alone (**Table 2**). Emerging nanodrug, physiotherapies, oncolytic viruses, and other treatments, although not yet approved for clinical medicine, have shown excellent results in animal studies. These treatments are likely to play essential roles in the next generation of oncology treatment strategies.

**Table 2:** Clinical trials targeting pyroptosis to enhance ICB therapy

NCT number	Study name	Treatment strategies	Cancer type
NCT03349710	Nivolumab or Nivolumab Plus Cisplatin, in Combination With Radiotherapy in Patients With Cisplatin-ineligible or Eligible Locally Advanced Squamous Cell Head and Neck Cancer	Biological: nivolumab Drug: cetuximab Drug: cisplatin Radiation: radiotherapy	Ovarian cancer
NCT03143153	A Study to Evaluate Efficacy in Subjects With Esophageal Cancer Treated With Nivolumab and Ipilimumab or Nivolumab Combined With Fluorouracil Plus Cisplatin Versus Fluorouracil Plus Cisplatin (CheckMate 648)	Biological: nivolumab Biological: ipilimumab Drug: cisplatin Drug: fluorouracil	Various advanced cancer
NCT01454102	Study of Nivolumab (BMS-936558) in Combination With Gemcitabine/Cisplatin, Pemetrexed/Cisplatin, Carboplatin/Paclitaxel, Bevacizumab Maintenance, Erlotinib, Ipilimumab or as Monotherapy in Subjects With Stage IIIB/IV Non-small Cell Lung Cancer (NSCLC) (CheckMate 012)	Biological: nivolumab Drug: gemcitabine Drug: cisplatin Drug: pemetrexed Drug: paclitaxel Drug: carboplatin Drug: bevacizumab Drug: erlotinib Biological: ipilimumab	Non-small cell lung cancer
NCT01450761	Trial in Extensive-Disease Small Cell Lung Cancer (ED-SCLC) Subjects Comparing Ipilimumab Plus Etoposide and Platinum Therapy to Etoposide and Platinum Therapy Alone	Biological: ipilimumab Biological: placebo matching ipilimumab Drug: etoposide Drug: cisplatin Drug: carboplatin	Small cell lung carcinoma
NCT03348904	Nivolumab and Epacadostat With Platinum Doublet Chemotherapy Versus Platinum Doublet Chemotherapy in Non-Small Cell Lung Cancer	Drug: nivolumab Drug: epacadostat Drug: placebo Drug: carboplatin Drug: cisplatin Drug: gemcitabine Drug: paclitaxel Drug: pemetrexed	Lung cancer
NCT02659059	Nivolumab in Combination With Ipilimumab (Part 1); Nivolumab Plus Ipilimumab in Combination With Chemotherapy (Part 2) as First Line Therapy in Stage IV <softenter>Non-Small Cell Lung Cancer (CheckMate 568)	Biological: nivolumab Biological: ipilimumab Drug: platinum doublet chemotherapy	Non-small cell lung cancer
NCT02041533	An Open-Label, Randomized, Phase 3 Trial of Nivolumab Versus Investigator’s Choice Chemotherapy as First-Line Therapy for Stage IV or Recurrent PD-L1+ Non-Small Cell Lung Cancer (CheckMate 026)	Biological: nivolumab Drug: gemcitabine Drug: cisplatin Drug: carboplatin Drug: paclitaxel Drug: pemetrexed	Stage IV or recurrent non-small cell lung cancer
NCT03215706	A Study of Nivolumab and Ipilimumab Combined With Chemotherapy Compared to Chemotherapy Alone in First Line NSCLC (CheckMate 9LA)	Biological: ipilimumab Biological: nivolumab Drug: carboplatin Drug: paclitaxel Drug: pemetrexed Drug: cisplatin	Non-small cell lung cancer
NCT02899299	Study of Nivolumab Combined With Ipilimumab Versus Pemetrexed and Cisplatin or Carboplatin as First Line Therapy in Unresectable Pleural Mesothelioma Patients (CheckMate743)	Biological: nivolumab Biological: ipilimumab Drug: pemetrexed Drug: cisplatin Drug: carboplatin	Mesothelioma
NCT03349710	Nivolumab or Nivolumab Plus Cisplatin, in Combination With Radiotherapy in Patients With Cisplatin-ineligible or Eligible Locally Advanced Squamous Cell Head and Neck Cancer	Biological: nivolumab Drug: cetuximab Drug: cisplatin Radiation: radiotherapy	Squamous cell carcinoma of the head and neck
NCT02367781	A Study of Atezolizumab in Combination With Carboplatin Plus (+) Nab-Paclitaxel Compared With Carboplatin+Nab-Paclitaxel in Participants With Stage IV Non-Squamous Non-Small Cell Lung Cancer (NSCLC) (IMpower130)	Biological: atezolizumab Drug: carboplatin Drug: nab-paclitaxel Drug: pemetrexed	Carcinoma, non-squamous non-small cell lung cancer
NCT02366143	A Study of Atezolizumab in Combination With Carboplatin Plus (+) Paclitaxel With or Without Bevacizumab Compared With Carboplatin+ Paclitaxel+ Bevacizumab in Participants With Stage IV Non-Squamous Non-Small Cell Lung Cancer (NSCLC) (IMpower150)	Biological: atezolizumab Drug: bevacizumab Drug: carboplatin Drug: paclitaxel	Carcinoma, non-small cell lung cancer
NCT02578680	Study of Pemetrexed+Platinum Chemotherapy With or Without Pembrolizumab (MK-3475) in Participants With First Line Metastatic Nonsquamous Non-small Cell Lung Cancer (MK-3475-189/KEYNOTE-189)	Biological: pembrolizumab Drug: cisplatin Drug: carboplatin Drug: pemetrexed Dietary supplement: folic acid Dietary supplement: vitamin b12 Drug: dexamethasone Drug: saline solution	Non-small cell lung carcinoma

## Conclusions and perspectives

In summary, pyroptosis is a type of proinflammatory necrotic cell death mediated by the GSDM superfamily. Recent research has increased knowledge regarding the mechanisms and pathophysiological roles of pyroptosis, and revealed its roles in tumor development and therapy. Pyroptosis-associated therapy directly kills cancer cells and enhances antitumor immunity by releasing antigens, DAMPs, and cytokines. Pyroptosis in cancer is a type of ICD that profoundly and extensively affects the tumor immune microenvironment, and can potentially change tumors from “cold” to “hot.” The exploitation of pyroptosis, particularly combined with ICB treatment, shows promise for cancer treatment.

Although several strategies of pyroptotic therapy have yielded satisfactory outcomes, the results are difficult to generalize, because GSDMs are downregulated in many tumors^115^. Evidence suggests that epigenetic mechanisms play an important role in the regulation of pyroptosis. Critical molecules in pyroptosis, such as caspase and GSDM, are downregulated by methylation. Demethylating drugs, such as decitabine, promote cell pyroptosis^79^. Histone acetylation may also be involved in the regulation of pyroptosis. Several epigenetic drugs are already used in clinical oncology treatment, and many nanomedicines involving epigenetics are under development^116^. Elucidating the epigenetic mechanisms in pyroptosis will be a focus of future studies.

The benefits and disadvantages of pyroptosis in the tumor immune microenvironment should be further evaluated. Although many mechanisms have been proposed to explain the relationship between pyroptosis and tumor therapy, a complete understanding of the influence of pyroptosis on the tumor immune microenvironment is lacking. Current limited knowledge has indicated that the role of pyroptosis in tumors is complex, there are a lot of pros and cons of pyroptosis^76^. Research has suggested that high expression of GSDMB in breast cancer is associated with a low survival rate and results in a higher propensity for metastasis^117^. In non-small cell lung cancer, higher expression of GSDMD is associated with giant tumors and more advanced cancer stages. Pyroptosis is also associated with many adverse effects of cancer therapy, such as cytokine release syndrome in CAR T cell therapy^39,118^ or chemotherapy drug damage to normal tissues in chemotherapy^35^. Additionally, pyroptosis induces the secretion of proinflammatory cytokines, and chronic inflammation has traditionally been known to promote tumorigenesis. A comprehensive understanding of the role of pyroptosis in cancer may contribute to developing strategies for cancer treatment.

Notably, other types of cell death, such as necroptosis or ferroptosis, can induce an immune response and therefore may have synergistic roles in ICI treatment^11,46^. Both necroptosis and ferroptosis are forms of PCD that activate host antitumor immunity^119^. Tang et al.^11^ have summarized the roles of ferroptosis, necroptosis, and pyroptosis in anticancer immunity. Some existing cancer treatments, such as chemotherapy and radiotherapy, cause necroptosis or ferroptosis, thus showing potential for combination therapy with ICIs^11^. The crosstalk between PCD pathways also should be considered. Research on this crosstalk has led to the discovery of PANoptosis^13^. In-depth exploration of the molecular mechanisms of these forms of PCD and discussion of their feasibility as clinical therapeutic targets should lead to new approaches for tumor immunotherapy.

The advent of ICB has renewed hopes for cancer therapy. Integrated studies on pyroptotic cell death induction are expected to extend the applicability range and enhance the antitumor immunotherapeutic effects. Combination therapy with ICB plus pyroptosis-induced therapy holds promise in cancer therapy. Preclinical research is necessary to design active targeting agents, and determine safe doses and optimal strategies to maximize potential benefits while minimizing adverse effects of treatments.
